# Effects of social support on vision-related quality of life in older adults with dry eye disease: the chain mediating role of illness perception and coping style

**DOI:** 10.3389/fpsyg.2024.1411661

**Published:** 2024-07-19

**Authors:** Haoran Pan, Xubin Pan, Danfeng Gu, Xiaobo Wang

**Affiliations:** ^1^Department of Ophthalmology, Affiliated Hospital of Jiangnan University, Wuxi, Jiangsu, China; ^2^School of Wuxi Medical College, Jiangnan University, Wuxi, Jiangsu, China; ^3^Nursing Department, Affiliated Hospital of Jiangnan University, Wuxi, Jiangsu, China

**Keywords:** dry eye disease, vision-related quality of life, social support, illness perception, coping style, chain mediating effect

## Abstract

**Objective:**

This study explored the effects of social support, illness perception, coping style, and vision-related quality of life (VRQOL) in older patients with dry eye disease (DED) using a chain mediation model.

**Methods:**

A total of 407 patients with DED from a tertiary hospital in Wuxi, Jiangsu Province, China, between June and December 2023 were selected as participants. A demographic questionnaire, the Social Support Rating Scale, the Brief Illness Perception Questionnaire, the Medical Coping Modes Questionnaire, and the National Eye Institute Visual Functioning questionnaire-25 were all given to them to complete. IBM SPSS (version 27.0) was used for data analysis, and Model 6 of the PROCESS Macro was used to test the predicted chain mediation model.

**Results:**

The positive association between social support and VRQOL demonstrated the mediation role of illness perception and coping style. Social support affected VRQOL via three pathways: illness perception (effect = 0.190), confrontational coping style (effect = 0.103), and a combination of illness perception and confrontational coping style (effect = 0.067), accounted for 23.60%, 12.80%, and 8.32% of the total effect, respectively.

**Conclusion:**

Social support in older patients with DED can significantly and positively predict the VRQOL. In addition to the independent mediating effect of illness perception and confrontational coping style, a chain-mediating effect exists between social support and VRQOL. The study serves as a valuable strategy for healthcare professionals to prevent and intervene in VRQOL for older patients with DED in the future.

## 1 Introduction

Dry eye disease (DED) is the most common chronic ocular surface disease and is caused by inflammation of the ocular surface, tear instability, hyperosmolarity, and disorders related to the nervous system (Hakim and Farooq, 2022). Age-related increases in DED incidence have been observed, with previous research indicating that 34.4% of patients with DED in China may be older than 60 years (Zhang et al., 2020). As the Chinese population ages and life expectancy rises, DED will remain one of the leading causes of ophthalmic illness (Song et al., 2018; Cai et al., 2022). Patients typically experience various symptoms, such as photophobia, fatigue, itchiness, burning, and visual disturbance, and some may even experience severe visual problems and blindness in the later stages if treatment is delayed (Boboridis et al., 2023). Meanwhile, those with DED must bear a substantial economic burden and develop psychological problems such as anxiety and depression during prolonged therapy, making DED an important public health problem (Chan et al., 2021; Yu et al., 2022). Furthermore, the ongoing deterioration of visual function in older adults leads to inconvenience in daily behaviors and activities, threatening the ocular health and vision-related quality of life (VRQOL) of older patients with DED (Wang et al., 2020).

VRQOL is a multidimensional measure that reflects the impact of ocular diseases and their treatment and care on patients’ activities of daily living, mental health status, and social functioning (Morthen et al., 2022). The focus of DED therapy is gradually shifting from treating symptoms of ocular discomfort alone to restoring or at least enhancing VRQOL as health concepts and medical models evolve (Guo and Akpek, 2020). In clinical practice, DED has been found to be associated with significant decreases in multiple dimensions of VRQOL, and its effect on VRQOL is comparable to that of severe fundus illness (Li et al., 2012; Le et al., 2014; Wu et al., 2021). A few population-based research on VRQOL and DED have shown that DED will reduce work efficiency; increase the risk of unemployment; disrupt normal activities such as reading, writing, and cooking; as well as increasing the burden of daily life (Dana et al., 2020; Morthen et al., 2023). It also increases the risk of falls and injuries among older adults (Barabino, 2022). Moreover, a study revealed that patients with DED over 50 years of age had lower VRQOL scores than patients younger than 50 (Paulsen et al., 2014). It can be seen that patients’ daily lives are significantly impacted by DED, especially those of older patients. Therefore, health professionals must take reasonable measures to enhance the VRQOL of older patients with DED.

Social support is a useful and effective external resource that provides individuals with the assistance they require while under strain or facing difficulties (Shen et al., 2022). Social support plays an essential role in the physical and mental health of older patients and is a predictor of their healthy outcome (Zheng et al., 2022). According to previous research, higher subjective happiness levels in DED patients are associated with a lower self-reported incidence of dry eye symptoms (Kawashima et al., 2015). Additionally, supportive social relationships can help alleviate negative emotions in the treatment of DED patients, including anxiety and worry (Viet Vu et al., 2019). This may indirectly explain the positive effects of social support on DED patients’ mental and physical wellbeing. However, no studies have directly examined the influence of social support on VRQOL in older patients with DED; this is important, as according to social support theory, excellent social support promotes individual health (Hupcey, 1998). Thus, this study proposes:

*H1*: social support positively predicts the VRQOL of older patients with DED.

Illness perception is a possible mediator in the association between social support and VRQOL in older patients with DED. Illness perception is a psychological concept based on Leventhal et al. (2016)’s common sense model of self-regulation (CSM), which describes a process in which patients recognize and evaluate diseases and symptoms using their knowledge and experience. Illness perception is an important index for predicting the quality of life of patients with chronic diseases, as many studies have reported. According to a cross-sectional study, those with glaucoma who had a negative illness perception and thought they had more eye-related symptoms were more likely to have poorer VRQOL (Zhang et al., 2022). One study investigating 706 patients with DED from five European nations found that they had severely negative cognition of DED. Indeed, negative illness perception contributes to unfavorable health outcomes (Labetoulle et al., 2017). Furthermore, insufficient social networks in patients with age-related eye disease were independently associated with ongoing psychological problems (e.g., anxiety and depression); we thought that this might negatively affect the patients’ perception of their situation (Hernández-Moreno et al., 2021; Zhang et al., 2024). Therefore, this study proposes:

*H2*: illness perception mediates the relationship between social support and VRQOL.

Coping styles are the cognitive approaches and behavioral strategies that people turn to in the face of stress and frustration and provide another psychological process that may affect VRQOL (Sturrock et al., 2015). Research has demonstrated that coping style predicts individual healthy activities and those who have effective coping styles report having fewer mental and physical impairments and engage in more healthy behaviors (Knowles et al., 2023; Chen et al., 2024). Throughout the treatment and rehabilitation of older patients with DED, a positive coping style can help them confront their illness more proactively and develop long-term resilience. In addition, some studies in health psychology have shown that social support can affect patients’ health outcomes and disease prognosis by stimulating them to adopt a corresponding coping style (Tian et al., 2021; Luo et al., 2023). Based on these findings, this study proposes:

*H3*: coping style mediates the relationship between social support and VRQOL.

Furthermore, according to the CSM, when patients perceive threats to their health, their subjective views may change their coping strategies, which may in turn affect their health outcomes (Leventhal et al., 2016). Several studies have supported the influence of illness perception and coping style on the quality of life of individuals with chronic illnesses (Tu et al., 2022; O'Connor et al., 2023). Thus, this study proposes:

*H4*: illness perception and coping style play a chain-mediating role between social support and VRQOL.

**Figure 1:**
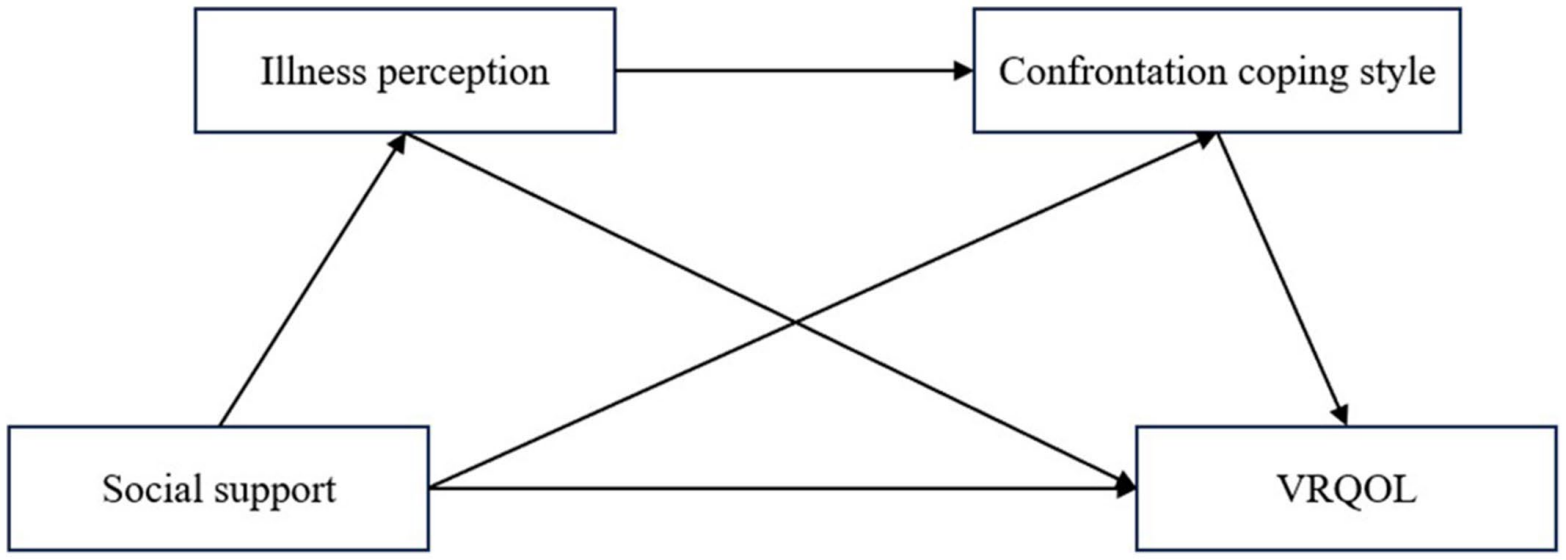
Hypothesized model. VRQOL, vision-related quality of life.

However, the association among social support, illness perception, coping style, and VRQOL in older patients with DED is unclear. The purpose of this study was to investigate and analyzed the correlations between these four variables. The hypothesized conceptual framework is shown in Figure 1, which aims to provide valuable evidence for establishing targeted interventions to improve VRQOL in older patients with DED.

## 2 Methods

### 2.1 Participants and setting

The survey was conducted at a general tertiary hospital in Wuxi, Jiangsu Province, southern China, between June and December 2023. A total of 410 questionnaires were distributed; three invalid questionnaires were excluded, while 407 valid questionnaires were retained. Thus, the effective response rate was 99.3%. Inclusion criteria: (1) meets the diagnostic criteria reported by TFOS DEWS II (Wolffsohn et al., 2017); (2) is aged 60 years or older; (3) voluntary signing of informed consent; and (4) have clear consciousness and can cooperate with the investigator. Exclusion criteria: (1) presence of any other chronic eye disease that might affect the ocular surface; (2) active eye lesions or eye surgery within the past 3 months; and (3) with severe psychological and mental problems as assessed by a psychiatrist.

### 2.2 Data collection

The study followed the principles of the Declaration of Helsinki. All procedures were approved by the Ethics Committee of the Affiliated Hospital of Jiangnan University (LS2024031). Participants were informed of the study’s purpose and significance, and the questionnaires were completed anonymously after signing informed consent. For anybody who had difficulty reading or writing, the investigators filled out the questionnaire based on the patients’ verbal responses. The investigators were nursing staff from the ophthalmology department who had received professional training. To protect the respondents’ privacy, each completed questionnaire was gathered and sealed.

### 2.3 Measures

#### 2.3.1 Demographic and clinical characteristics

The participants’ demographic data included age, gender, occupation, marital status, place of residence, educational level, medical insurance type, monthly family income (in Chinese yuan), disease duration, smoking history, and clinical data such as treatment condition, disease health education, and comorbidities.

#### 2.3.2 Questionnaires

##### 2.3.2.1 Social support rating scale (SSRS)

The Social Support Rating Scale (SSRS) was originally developed by the Chinese scholar Xiao (1994). It has three dimensions and 10 items, including objective support, subjective support, and the utilization of support. A 4-point Likert scale was adopted, with a total score between 12 and 66 points; higher scores indicated greater levels of social support. China has made extensive use of the SSRS, with a Cronbach’s α of 0.920. The scale’s Cronbach’s α in this study was 0.878.

##### 2.3.2.2 Brief illness perception questionnaire (BIPQ)

The Brief Illness Perception Questionnaire (BIPQ) was designed by Broadbent et al. (2006). It consists of 9 items across three dimensions: illness comprehensibility, illness cognitive representations, and emotional representations. The scores range from 0 to 80, and the final item is open-ended, so it does not contribute to the overall mark. Patients with higher scores indicate that the illness is perceived as more threatening. In the current study, the Cronbach’s α was 0.859.

##### 2.3.2.3 Medical coping modes questionnaire (MCMQ)

The Medical Coping Modes Questionnaire (MCMQ) was designed by Feifel et al. (1987). It has been verified that the Chinese version of the instrument has strong validity and reliability. The questionnaire has 20 items across three dimensions: confrontation (8 items), avoidance (7 items), and resignation (5 items). The total score is 20–80, and the three dimensions range from 8 to 32, 7 to 28, and 5 to 20, respectively. Patients are more likely to use this coping style if they average score better on one dimension, with respective Cronbach’s α of 0.838, 0.763, and 0.860 in this study.

##### 2.3.2.4 National eye institute visual functioning questionnaire-25 (NEI-VFQ-25)

The National Eye Institute Visual Functioning Questionnaire- 25 (NEI-VFQ-25) was simplified by Mangione et al. based on the NEI-VFQ-51 (Mangione et al., 2001). The scale has 12 dimensions: general health, general vision, ocular pain, near activities, distance activities, social functioning, mental health, role difficulties, dependency, driving, color vision, and peripheral vision. This study evaluated VRQOL using the Chinese version of the NEI-VFQ-25. The score ranges from 0 to 100; the lower the score, the worse the patients’ VRQOL, accompanied by severe visual impairment. In addition, Cronbach’α for the scale in this study was 0.956. Notably, in China and other Asian countries, the missing rate of the driving dimension is high, and its applicability to older adults is low. Therefore, this study removed those dimensions following suggestions for questionnaire usage (Le et al., 2012).

### 2.4 Statistical analysis

All data were analyzed using SPSS version 27 (IBM, Inc., Armonk, NY, USA). In our study, all continuous variables met the assumptions of normality based on the Shapiro–Wilk test and linearity for path analysis. The demographic measurement data were described using the mean ± standard deviation (M ± SD), while categorical data were described statistically by frequency (n) and percentage (%). One-way analysis of variance and independent t-tests were used to assess the statistical significance of the various clinical and demographic categories. The association among social support, illness perception, coping style and VRQOL were examined using Pearson’s correlation analysis. Harman’s single-factor test was used to examine the possible standard deviation before data analysis and further improve the rigor of the research (Podsakoff et al., 2003). Model 6 of the PROCESS Macro was utilized to estimate the chain-mediating effect. Demographic and clinical characteristics, including age, gender, marital status, educational level, medical insurance type, DED duration, smoking history, DED treatment, and DED health education that can significantly affect the dependent variable (Table 1) were included as control variables into the model. Bootstrapping was used to assess the mediating effect’s significance using 5,000 random samples. The mediating impact was deemed statistically significant if there was no zero in the matching 95% bias-corrected confidence interval (CI). A *p*-values <0.05 were considered statistically significant.

**Table 1:** Demographic and clinical characteristics by VRQOL and results of the univariate model (*n* = 407).

Variables	VRQOL		*t / F*	*p*- value
	*N* (%)	M ± SD		
Age			11.668	<0.001
60–69	157 (38.57)	65.73 ± 18.87		
70–79	200 (49.14)	60.04 ± 15.90		
≥80	50 (12.29)	51.10 ± 19.55		
Gender			10.077	<0.001
Male	169 (41.52)	72.52 ± 14.59		
Female	238 (58.48)	54.47 ± 17.43		
Residence			1.947	0.144
Urban	202 (49.63)	62.90 ± 17.26		
Rural	102 (25.06)	59.75 ± 19.08		
Town	103 (25.31)	59.05 ± 18.60		
Marital status			2.220	0.027
Married	334 (82.06)	62.06 ± 17.72		
Single/Divorced/Widowed	73 (17.94)	56.89 ± 19.37		
Education level			18.787	<0.001
Primary and below	159 (39.07)	54.45 ± 20.24		
Middle school	109 (26.78)	61.09 ± 15.32		
High school	86 (21.13)	67.25 ± 14.21		
Junior college or above	53 (13.02)	71.35 ± 13.94		
Occupation			2.198	0.112
Inservice	110 (27.03)	64.13 ± 17.14		
Retire	216 (53.07)	60.34 ± 18.44		
Other	81 (19.90)	59.18 ± 18.23		
Monthly family income			2.378	0.069
<3,000 yuan	59 (14.50)	56.60 ± 18.96		
3,000 ~ 5,000 yuan	79 (19.41)	59.73 ± 18.22		
5,000 ~ 8,000 yuan	130 (31.94)	61.25 ± 16.80		
>8,000 yuan	139 (34.15)	63.75 ± 18.60		
Medical insurance			3.506	<0.001
Yes	338 (83.05)	62.55 ± 17.83		
No	69 (16.95)	54.22 ± 18.01		
Duration of DED			10.105	<0.001
<3 months	207 (50.86)	64.10 ± 17.01		
3–6 months	63 (15.48)	62.67 ± 19.98		
6–12 months	69 (16.95)	61.07 ± 15.23		
>12 months	68 (16.71)	50.74 ± 18.81		
Smoking history			−3.706	<0.001
Yes	208 (51.11)	57.93 ± 18.11		
No	199 (48.89)	64.48 ± 17.54		
DED related treatment			5.922	<0.001
Yes	198 (48.65)	66.38 ± 16.97		
No	209 (51.35)	56.17 ± 17.79		
DED related health education			8.772	<0.001
Yes	191 (46.93)	68.75 ± 15.01		
No	216 (53.07)	54.40 ± 17.98		
Comorbidities			1.595	0.111
No	126 (30.96)	63.27 ± 18.15		
Yes	281 (69.04)	60.18 ± 18.05		

M, mean; SD, standard deviation. VRQOL, vision-related quality of life.

## 3 Results

### 3.1 Common methods bias test

Applying Harman’s single-factor test to identify common methodological deviations. Nine variables with characteristic values larger than one were found in the results. The first component explained 38.07% of the variance, which was lower than the 40% critical criterion. Thus, this study showed no severe common method bias.

### 3.2 Participant characteristics

The mean age of the 407 patients was 71.63 (±6.19) years, with a range of 60 to 90 years. Univariate results for the sample population and their demographics are shown in Table 1. Over half of the patients (58.5%) were female, 82.10% were married, and 49.6% resided in urban areas. A total of 34.2% had a high school degree or higher and approximately 53.1% were retired. Over half of the participants (66.1%) had a monthly family income of more than 50,00 yuan, and the majority (83.0%) had health insurance. Among these patients, 51.10% were previously smokers. Over half (50.9%) of the patients had DED for less than 3 months at the time of the survey. Approximately 48.7% of patients received suitable therapy, whereas 46.9% received related health education. Two-thirds of the patients had at least one comorbidity (69.0%). Furthermore, residence, occupation, monthly family income, and comorbidities had no statistically significant relationship with VRQOL. Other characteristics were statistically significant with VRQOL (*p* < 0.05) and were included as control variables in the multiple linear regression model.

### 3.3 Variable information

Table 2 demonstrates the mean and standard deviation of the different variables with a mean score of 61.14 (±18.11), the VRQOL for the entire study population was classified as moderate. The social support score was 38.53 (±11.54), indicating a medium level of social support. Patients perceived illness as a threat, as indicated by their mean illness perception score of 50.39 (±10.73). The scores for the confrontation, avoidance, and resignation coping styles were 18.94 (±4.54), 16.04 (±3.85), and 11.98 (±3.65), while the average scores were 2.37 (±0.57), 2.29 (±0.55), and 2.41 (±0.75), respectively. Based on their average scores, the patients tended to adopt a resignation coping style.

**Table 2:** Mean and standard deviations among the variables (*n* = 407).

Variables	M ± SD
VRQOL	61.14 ± 18.11
Social support	38.53 ± 11.54
Illness perception	50.39 ± 10.73
Confrontation	18.94 ± 4.54
Avoidance	16.04 ± 3.85
Resignation	11.98 ± 3.65

M, mean; SD, standard deviation. VRQOL, vision-related quality of life.

### 3.4 Correlation analysis

Table 3 indicates a significant positive correlation between VRQOL and both social support (*r* = 0.713, *p* < 0.001) and confrontational coping style (*r* = 0.664, *p* < 0.001). Conversely, VRQOL was found to have a negative relationship with illness perception (*r* = −0.703, *p* < 0.001) and resignation coping style (*r* = −0.720, *p* < 0.001). Additionally, social support was positively correlated with confrontational coping style (*r* = 0.562, *p* < 0.001) and negatively correlated with resignation coping style (*r* = −0.581, *p* < 0.001) and illness perception (*r* = −0.707, *p* < 0.001). Furthermore, illness perception had a positive correlation with resignation coping style (*r* = 0.579, *p* < 0.001) and a negative correlation with confrontational coping style (*r* = −0.580, *p* < 0.001). No correlations were observed between avoidance coping style and the other key variables.

**Table 3:** Correlation between social support, illness perception, coping style, and VRQOL (*n* = 407).

Variables	VRQOL	Social support	Illness perception	Confrontation	Avoidance	Resignation
VRQOL	1.000	-	-	-	-	-
Social support	0.713*	1.000	-	-	-	-
Illness perception	−0.703*	−0.707*	1.000	-	-	-
Confrontation	0.664*	0.562*	−0.580*	1.000	-	-
Avoidance	−0.044	−0.072	0.011	−0.163*	1.000	-
Resignation	−0.720*	−0.581*	0.579*	−0.589*	−0.029	1.000

*p<0.001. VRQOL, vision-related quality of life.

### 3.5 Mediation analysis

**Figure 2:**
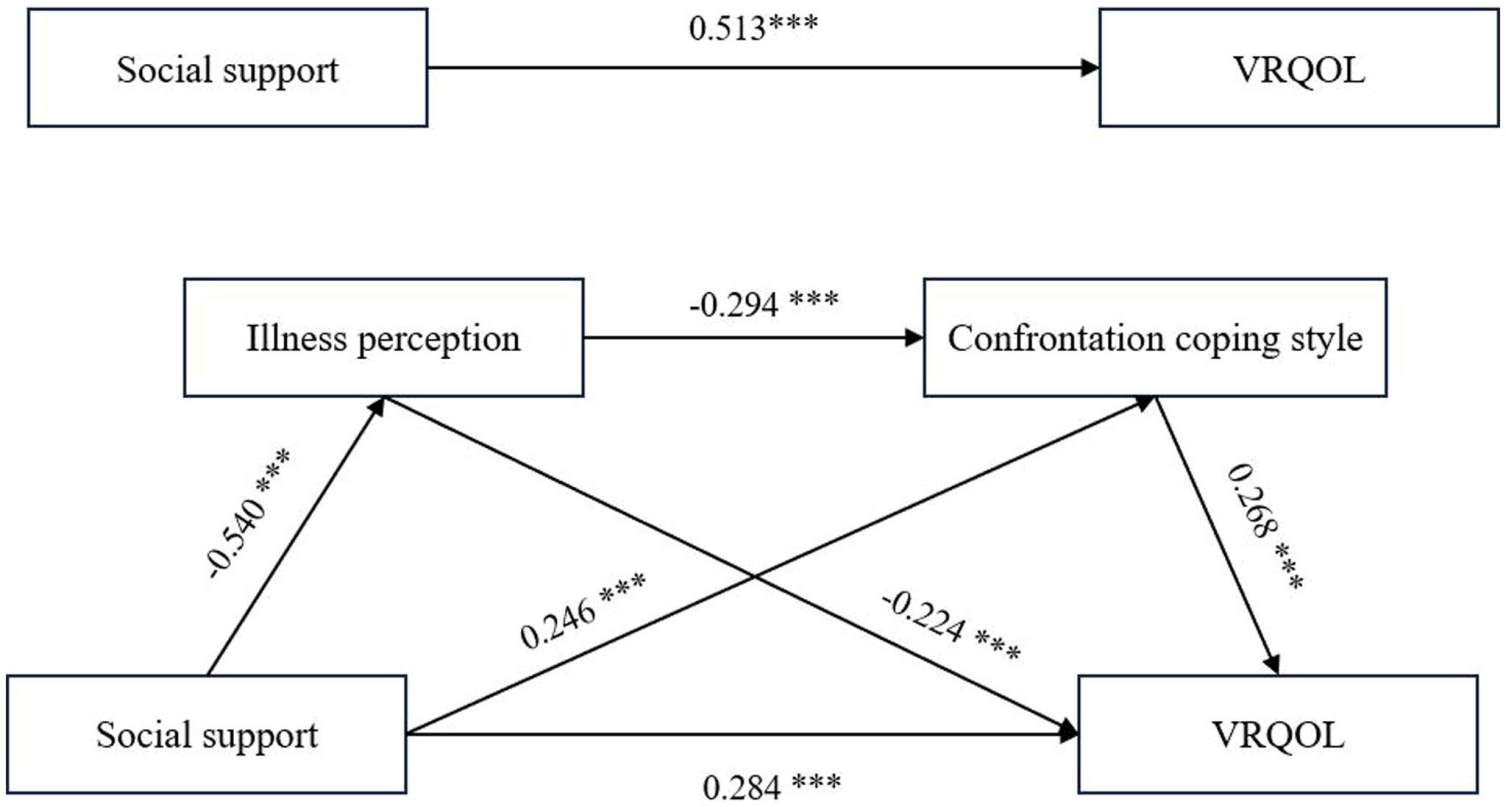
The chain-mediating model of illness perception and confrontation coping style in the relationship between social support and VRQOL. ***p < 0.001 was considered statistically significant. VRQOL, vision-related quality of life.

This study assessed the chain-mediation impact with the Model 6 of the PROCESS Macro. In the study, social support was examined as the independent variable, while illness perception, confrontational coping style, and resignation coping style were viewed as the intermediary variable, with VRQOL being the outcome variable. Figure 2 and Table 4 show the correlation between illness perception and confrontational coping style on social support and VRQOL after controlling for nine variables (e.g., age, gender, marital status, educational level).

**Table 4:** Regression model of the relationship between factors (*n* = 407).

Variables	VRQOL		Illness perception		Confrontation		VRQOL	
	*β*	*t*	*β*	*t*	*β*	*t*	*β*	*t*
Age	−0.082	−2.475*	0.040	1.165	−0.070	−1.757	−0.051	−1.715
Gender	−0.127	−3.545***	0.126	3.373*	−0.018	−0.426	−0.084	−2.576*
Marital status	−0.020	−0.627	0.012	0.349	−0.009	−0.235	−0.014	−0.493
Education level	0.090	2.623**	−0.025	−0.704	0.071	1.733	0.064	2.052*
Duration of DED	−0.083	−2.521*	0.078	2.268*	−0.058	−1.474	−0.044	−1.472
Smoking history	0.087	2.660**	−0.053	−1.548	0.020	0.514	0.065	2.232*
treatment	−0.077	−2.266*	0.080	2.261*	0.029	0.703	−0.060	−1.971*
health education	−0.148	−4.293***	0.113	3.150*	−0.155	−3.734***	−0.072	−2.264*
Medical insurance	−0.021	−0.640	0.078	2.274*	−0.031	−0.787	0.011	0.368
Social support	0.513	12.728***	−0.540	−12.837***	0.246	4.296***	0.284	6.454***
Illness perception					−0.294	−5.118***	−0.224	−5.028***
Confrontation							0.268	7.093***
*R^2^*	0.594		0.557		0.423		0.676	
*F*	57.982***		49.834***		26.319***		68.521***	

*p < 0.05, **p < 0.01, ***p < 0.001. VRQOL, vision-related quality of life.

Multiple linear regression analysis revealed that social support had a significant positive total effect on VRQOL (*β* = 0.513, *p* < 0.001). It directly affected VRQOL (*β* = 0.284, *p* < 0.001), while also negatively predicting illness perception (*β* = −0.540, *p* < 0.001) and positively predicting confrontational coping style (*β* = 0.246, *p* < 0.001). Meanwhile, illness perception had a negative effect on both VRQOL (*β* = −0.224, *p* < 0.001) and confrontational coping style (*β* = −0.294, *p* < 0.001). Confrontational coping style positively predicted VRQOL (*β* = 0.268, *p* < 0.001). The results for illness perception and resignation coping style as mediating variables were not statistically significant.

### 3.6 Bootstrap tests

Bootstrap tests were repeated 5,000 times to test the mediation effect. The mediation effect was considered significant because the 95% bootstrap confidence interval of the effect did not reach zero. As Table 5 shows, the direct effect pathway was social support → VRQOL, the effect value is 0.445 (95% CI: 0.310 to 0.581), which accounts for 55.28% of the total effect.

**Table 5:** Bootstrap analysis of the mediating effect of social support and VRQOL (*n* = 407).

Paths	Effect	Boot SE	Boot LL CI	Boot UL CI	Effect ratio
Total effect	0.805	0.063	0.681	0.930	100.00%
Direct effect	0.445	0.069	0.310	0.581	55.28%
Total indirect effect	0.360	0.048	0.266	0.455	44.72%
Indirect effect 1	0.190	0.044	0.106	0.277	23.60%
Indirect effect 2	0.103	0.026	0.054	0.157	12.80%
Indirect effect 3	0.067	0.019	0.033	0.106	8.32%

Indirect effect 1: Social support →Illness perception → VRQOL; Indirect effect 2: Social support →Confrontation → VRQOL; Indirect effect 3: Social support →Illness perception →Confrontation → VRQOL. VRQOL, vision-related quality of life.

The total indirect effect was 0.360 (95% CI: 0.266 to 0.455), which accounted for 44.72% of the total effect. Three mediatory impact pathways were identified: social support → illness perception → VRQOL, with an indirect effect value of 0.190 (95% CI: 0.106 to 0.277), accounting for 23.60% of the total effect; social support → confrontation → VRQOL, with an indirect effect value of 0.103 (95% CI: 0.054 to 0.157), accounting for 12.80% of the total effect; and social support → illness perception → confrontation → VRQOL, with an indirect effect value of 0.067 (95% CI: 0.033 to 0.106), accounting for 8.32% of the total effect.

## 4 Discussion

This study concentrated on the mediating effects of social support and VRQOL in older patients with DED. Social support was found to promote VRQOL through the indirect pathways of illness perceptions and confrontational coping style, as well as the chain mediation path of illness perceptions and confrontational coping style.

According to this study’s results, the mean VRQOL score of older patients with DED is 61.14 (±18.11), which is lower than the results of 71.60 (±12.80) points by Hossain et al. (2021) and 83.0 (±9.0) points by Erøy et al. (2023). This discrepancy could be clarified by the fact that the studies were conducted at various locations on different dates. Furthermore, the participants in this study were older patients whose visual function was worse than that of the adult patients in previous studies. Older patients with DED generally suffer from the disease for a long time and recover slowly, which leads to increased psychological stress. Additionally, their ocular symptoms result in decreased visual function and, to some extent, limit their independence, which lowers VRQOL.

This study’s results showed that social support had a positive predictive effect on the VRQOL of older patients with DED (Hypothesis 1). Older patients with DED have greater health outcome when they have more social support, which agree with the results of several similar studies examining patients with glaucoma (Uruthiramoorthy et al., 2020) and diabetic retinopathy (Zhang et al., 2024). Social support is well-known to be a significant external component. Nevertheless, the mean social support score of older patients with DED in this study was moderate 38.53 (±11.54). Patients with a sufficiently large social network can receive practical, emotional, and interpersonal support in addition to health assistance, which helps improve their health and lessen the negative effects of illness. For example, a qualitative study revealed that patients with DED are more willing to spend more time socializing with family and friends because it helps them divert their attention and reduce the painful impact of their eye symptoms on their lives (Yeo and Tong, 2018). Therefore, health professionals and families should encourage patients to participate in as many activities as possible to broaden their social network system and obtain appropriate support and assistance to promote good health outcomes and increase their VRQOL.

In addition, this study’s results revealed that illness perception mediated the relationship between social support and VRQOL in older patients with DED (Hypothesis 2). The mean illness perception score for participants in this study was high 50.39 (±10.73). The results align with a previous study (Dunn et al., 2021), which also emphasized that DED patients lack a correct understanding of the disease; more knowledge and education regarding the etiology of DED and therapeutic approaches are essential for changing patients’ perceptions. Social support from family, friends, and the external environment can stimulate patients’ latent cognitive and psychological attributes and help them develop an accurate perception of the disease (Segal et al., 2021). Positive illness perception can effectively establish reasonable beliefs and improve physical and mental health, thereby enhancing the overall quality of life for older patients with DED. Sano et al. (2018) discovered that educating participants with DED on how exercise can alleviate eye symptoms and promote health over a 10-week period resulted in improved subjective symptoms of DED. Consequently, healthcare professionals should instruct patients to use the various social resources available to them to obtain appropriate knowledge and skills related to the disease, as well as guide them in developing a positive attitude toward coping with the disease to maintain a good emotional state and, ultimately, improve VRQOL.

Finally, this study’s results demonstrated that a confrontational coping style mediated the relationship between social support and VRQOL in older patients with DED (Hypothesis 3). This study found that older patients with DED were more likely to adopt a resignation coping style. DED is a disease that requires long-term and repeated physical therapy and daily eye drops, which reduces patients’ self-confidence in recovering from the disease, leads to a pessimistic attitude, and causes them to cope with the disease by succumbing to it. However, when people have more social support, they can fully mobilize social resources in the face of the threat of disease, take the initiative to learn about the disease, and cope with the challenges it presents (Zamanian et al., 2021). Patients with a high level of social support have a rich social network, usually have a constructive attitude toward the illness, a high level of adherence, and good health behaviors (Li et al., 2023; Yang et al., 2024). These actions result in the formation of positive coping strategies and health management behaviors, which, in turn, support patients’ physical and psychological recovery and enhance their VRQOL.

Notably, we also discovered that social support can affect VRQOL in older patients with DED through the chain mediation of illness perception and confrontational coping style (Hypothesis 4). Put simply, increasing social support levels for older patients with DED will help them positively perceive and cope with the disease and improve their VRQOL. The indirect effect ratio was 8.32%. With a high degree of social support, patients can obtain sufficient cognitive, emotional, and material motivation, which is not only conducive to improving their illness perception but also helps relieve negative feelings. The positive illness perception motivates patients to use constructive coping styles, improves their ability to handle stressful situations, and increases their VRQOL during the disease period. Overall, these findings offer fresh evidence about CSM in the context of older patients with DED. Our results emphasize how critical it is to better understand the underlying mechanisms to establish effective therapies for older patients with DED.

### 4.1 Limitations and recommendations

This study had some limitations. First, the samples were collected from a single hospital, more verification is needed to ensure that the study’s results are representative. Future research should involve multicenter large-sample analyses to verify our findings. Second, this study was cross-sectional, so we were unable to explain the dynamic development and changes between the variables. The causal relationships among these variables can be further investigated in the future using better methods such as longitudinal studies. Third, self-report questionnaires served as all of the instruments in this investigation; we also did not assess the patients’ cognitive level using a structure test (e.g., a mini-mental test); we only assessed it based on the judgment of the psychiatrists, which may be subjective. Thus, relatively objective methods can be combined to improve the study’s accuracy in the future. Fourth, considering we did not want to increase the burden on older patients, we did not measure their psychological status. In the future, it is necessary to explore the effects of psychological factors (e.g., anxiety and depression) on the VRQOL of DED patients to provide more evidence for the study of VRQOL. Finally, although this study included only older patients with DED, future studies should include patients of all ages.

## 5 Conclusion

As far as we are aware, this cross-sectional study is the first to investigate the association among social support, illness perception, coping styles, and VRQOL in older patients with DED. Social support has a positive effect on VRQOL in older patients with DED. Illness perception and confrontational coping style not only mediate the relationship between social support and VRQOL independently but also have chain mediating effects. Thus, this study provides a theoretical basis and reference for healthcare professionals to formulate effective intervention strategies to improve VRQOL in older patients with DED in the future.

## Data availability statement

The raw data supporting the conclusions of this article will be made available by the authors, without undue reservation.

## Ethics statement

The studies involving humans were approved by Affiliate Hospital of Jiangnan University. The studies were conducted in accordance with the local legislation and institutional requirements. The participants provided their written informed consent to participate in this study. Written informed consent was obtained from the individual(s) for the publication of any potentially identifiable images or data included in this article.

## Author contributions

HP: Writing – original draft, Writing – review & editing, Data curation, Investigation. XP: Funding acquisition, Resources, Supervision, Writing – review & editing. DG: Funding acquisition, Methodology, Resources, Supervision, Writing – review & editing. XW: Data curation, Investigation, Writing – original draft.
